# Pyroptosis Provides New Strategies for the Treatment of Cancer

**DOI:** 10.7150/jca.77965

**Published:** 2023-01-01

**Authors:** Yuming Jia, Xin Wang, Yanli Deng, Shengchao Li, Xiaowu Xu, Yi Qin, Li Peng

**Affiliations:** 1 Department of Hepatobiliary Surgery, The Fourth Hospital of Hebei Medical University, Shijiazhuang, China.; 2 Emergency Department, The Fourth Hospital of Hebei Medical University, Shijiazhuang, China.; 3 Department of Clinical Laboratory, The Fourth Hospital of Hebei Medical University, Shijiazhuang, China.; 4 Department of Pancreatic and Hepatobiliary Surgery, Fudan University Shanghai Cancer Center, Shanghai, China.; 5 Pancreatic Cancer Institute, Fudan University, Shanghai, China.

**Keywords:** pyroptosis, cancer, tumor immunity, gasdermin, tumor treatment

## Abstract

Cancer is an important cause of death worldwide. The main types of cancer treatment are still surgery, chemotherapy and radiotherapy, and immunotherapy is becoming an important cancer treatment. Pyroptosis is a type of programmed cell death that accompanies an inflammatory response. This paper reviews the recent research progress in pyroptosis in tumors. Pyroptosis has been observed since 1986 and until recently has been recognized as programmed cell death mediated by GSDM family proteins. The molecular pathway of pyroptosis depends on the inflammasome-mediated caspase-1/GSDMD pathway, which is the canonical pathway, and the caspase-4/5/11/GSDMD pathway, which is the noncanonical pathway. Other pathways include caspase3/GSDME. Pyroptosis is a double-edged sword that is closely related to the tumor immune microenvironment. On the one hand, pyroptosis produces a chronic inflammatory environment, promotes the transition of normal cells to tumor cells, helps tumor cells achieve immune escape, and promotes tumor growth and metastasis. On the other hand, some tumor cell treatments can induce pyroptosis, which is a nonapoptotic form of cell death. Additionally, pyroptosis releases inflammatory molecules that promote lymphocyte recruitment and enhance the immune system's ability to kill tumor cells. With the advent of immunotherapy, pyroptosis has been shown to enhance the antitumor efficacy of immune checkpoint inhibitors. Some antineoplastic agents, such as chemotherapeutic agents, can also exert antineoplastic effects through the pyroptosis pathway. Pyroptosis, which is a programmed cell death mode, has been the focus of research in recent years, and the relationship between pyroptosis, tumors and tumor immunity has attracted attention, but there are still some questions to be answered regarding the specific mechanism. Further study of pyroptosis would aid in developing new antitumor therapies and has great clinical prospects.

## Introduction

Cancer is an important cause of death worldwide and has gradually become a major global public health problem 1. Currently, surgery, radiotherapy and chemotherapy are the main forms of cancer treatment. The rise in immunotherapy in recent years offers new options for cancer treatment 2. Many of the ideas behind cancer treatment lie in inducing tumor cell death. Cell death is a complex and important regulatory network in which the immune system is also involved. Cell death can be divided into programmed death and nonprogrammed death. Programmed death includes apoptosis, necrotizing apoptosis, autophagy, and ferroptosis 3. Pyroptosis is a type of lytic inflammatory programmed cell death characterized by swelling and dissolution of cells and is accompanied by the release of various proinflammatory factors. As a form of programmed death, pyroptosis occurs more quickly and with a stronger inflammatory response than other forms of death. Recent studies have shown that pyroptosis is closely related to both tumors and immunity 4. The pyroptosis pathway is involved in both the innate and adaptive immune systems. The core of the pyroptosis pathway is the gasdermin (GSDM) protein family, which can be cleaved by caspases and granzymes to form active fragments. When activated, these proteins can cause membrane perforation, cell swelling and rupture, accompanied by the release of a large number of inflammatory factors, which then affect downstream pathways. Researchers have shown that pyroptosis may play dual roles in tumor activity. On the one hand, when normal cells are stimulated and pyroptosis occurs, inflammatory factors are released, leading to the formation of an inflammatory microenvironment, which can promote the transformation of normal cells into cancer cells. On the other hand, appropriate levels of pyroptosis help to maintain the stability of the extracellular environment, improve immune activity, and remove damage and pathogens to protect the host. Inducing pyroptosis in cancer cells may become a new therapeutic strategy to inhibit the development of cancer 5.

It is well known that the transformation, growth, invasion and metastasis of human cancer cells, as well as the response to treatment, are regulated by molecular signals. Cell death plays an indispensable role in the biological process of maintaining the normal homeostasis of the body and the rapid proliferation of tumor cells 6, 7. Cell death includes regulatory cell death (RCD) and accidental cell death (ACD). Common RCDs include apoptosis, necrotic apoptosis, ferroptosis, autophagy and pyroptosis. Programmed death is an internal death mechanism controlled by these molecular signals.

Ferroptosis is an iron ion dependent RCD, mainly caused by lipid peroxidation. Necrotic apoptosis is mainly mediated by cytokines (TNF-a, IFN-a and IFN-g), Toll like receptors (TLR3, TLR4 and TLR9) and nucleic acid (DNA and RNA) receptors. MLKL is the key molecule of necrotizing apoptosis 8, 9. For decades, apoptosis has been the main mode of cell death studied by people, but the failure to induce apoptosis is the main reason for failure of cancer treatment 10. Therefore, pyroptosis, as a non- apoptotic mechanism, may make up for the shortcomings of apoptosis in cancer treatment. At present, pyroptosis has become a hot research topic in the field of cancer.

## Timeline of the pyroptosis study

In 1986, Friedlander and colleagues found that anthrax lethal toxin induced rapid lysis and death in mouse macrophages, as well as the release of intracellular substances 11. In 1992, Zychlinsky and colleagues discovered the morphological characteristics of pyroptosis and its difference from apoptosis through shigella-induced macrophage infection, and this process was considered as programmed cell death mediated by Caspase-1, at that time the concept of pyroptosis did not exist 12. In 1999, D. Hersh and colleagues found that caspase-1 knockout blocked Shigella-induced cell death 13. In 2001, Cookson BT et al. defined programmed cell death with an inflammatory response as pyroptosis 14. In 2005, pyroptosis was redefined by Fink SL et al as programmed cell death mediated by caspase-1 in which cells undergo nuclear contraction, DNA rupture, swelling and the release inflammatory factors 15. In 2015, gasdermin D (GSDMD) was shown to be a key protein in the pyroptosis pathway, which can be cleaved by caspase-1/4/5/11 to exert an effect 16, 17. In 2016, Liu S reported that the GSDMD protein is usually in a state of self-inhibition. After being cleaved by the aforementioned caspases in a specific position, GSDMD-N domain could lead to cell membrane perforation and thus induce pyroptosis 18, 19. In 2017, Shi J et al redefined pyroptosis as programmed cell death mediated by GSDM family proteins 20. The GSDM protein family includes GSDMA, GSDMB, GSDMC, and GSDME/DFNA5, which also have membrane perforation activity. The Nomenclature Committee on Cell Death (NCCD) defined pyroptosis as regulatory cell death (RCD) that relies on perforation of the plasma membrane and is caused by members of the GSDM protein family, often (but not always) as a result of inflammatory caspase activation in 2018 21.

## Signaling pathways of pyroptosis

In the past few years, many studies on the pyroptosis pathway have been conducted. Pyroptosis is generally considered to be inflammatory caspase-induced cell death. The GSDM protein family plays a decisive role in pyroptosis. These proteins consist of two different N-terminal and C-terminal domains linked by a flexible junction region. In the absence of activated cleavage, the binding of the C-terminal to the N-terminal can inhibit the activity of the N-terminal 19. When the GSDM protein is cleaved, the released GSDM-N terminal forms oligomers, which can lead to pyroptosis through plasma membrane perforation 22. GSDMD-related pathways are the most researched and are classified into canonical and noncanonical pathways. The caspase-1-dependent pyroptosis pathway is the canonical pathway, while the bacterial toxin lipopolysaccharide (LPS) activates the human caspase-4/5 or mouse caspase-11 pathway, which is the noncanonical pathway 23. All of these pathways can activate certain caspases to cleave GSDMD, thus releasing the N-terminal domain of GSDMD and causing pyroptosis 24. Recent studies have shown that caspases that perform apoptosis as well as granzymes can also induce pyroptosis by cleaving GSDM proteins 25.

### The canonical pathway

The signal transduction of pyroptotic inflammasome activation relies on pattern recognition receptors (PRRs) that recognize pathogen-associated molecular patterns (PAMPs) and nonpathogen-related damage-associated molecular patterns (DAMPs). Toll-like receptor (TLR), intracellular nucleotide binding oligomeric domain (NOD)-like receptor (NLR) and AIM2-like receptor (ALR) are all pyroptosis-related PRRs. PRRs are receptors for danger signals and can be activated by many factors, including viruses, fungi, bacterial toxins, parasites, nucleic acids, crystalline substrates, certain drugs, silica, reactive oxygen species (ROS), and endogenous damage signals 26-29. NLRP3, which is the most common PRR, is activated through two steps: activation of K^+^/Ca^2+^ outflow and mitochondrial- and lysosomal-related damage. After these factors recognize relevant PAMPs and DAMPs, caspase-1 is activated 30-33. The inflammasome is assembled and recruits ASC adapters, and the NLR or AIM2 signaling domain connects to ASC 34. This binding in turn recruits caspase-1, leading to caspase-1 activation, which cleaves and activates pro-IL-8 and IL-1, which are released into the extracellular space to trigger an inflammatory response 35. Some researchers have also shown that NLRC4 can directly bind caspase-1 in the absence of ASC 36. GSDMD can be specifically cleaved by caspase-1 to play a role in downstream plasma membrane perforation 16. In general, inflammation-mediated pyroptosis has been classified as the canonical caspase-1-dependent inflammatory pathway 37.

### The noncanonical pathway

The noncanonical pathway differs from the canonical pathway in that it does not require an inflammasome to activate caspase-1. Gram-negative LPS can directly activate caspase-4/5 in humans or caspase-11 in mice. Activated caspase-4/5/11 can cleave GSDMD to produce the N-terminal domain, which perforates the plasma membrane and induces pyroptosis 38. In addition, LPS-activated caspase-11 opens pannexin-1 (a nonselective large protein channel) 39 and allows K^+^ efflux, which activates the NLRP3 inflammasome and induces caspase-1 activation and the canonical pyroptosis pathway 40, 41. This process promotes the activation and release of IL-1β and IL-18, whose activation is caused by caspase-1 but not caspase-4/5/11. The formation of pores is not confined to the cell membrane, other membranes within the cell, such as mitochondrial membranes, can also form pores 42. During inflammatory lung injury, the LPS-mediated caspase-11/GSDMD pathway induces mitochondrial pores, resulting in the release of mitochondrial DNA into endothelial cells, which triggers downstream molecular pathways.

### Other pyroptosis pathways

Studies have shown that caspase-3/6/8, which is associated with apoptosis, can also induce pyroptosis. Caspase-3 can induce GSDME-related pyroptosis under certain conditions, such as in the presence of TNF-α, high expression of GSDME or certain chemotherapeutic agents 43. Zheng et al. found that GSDME is a switch between apoptosis and pyroptosis induced by chemotherapy drugs. Pyroptosis occurs when GSDME is highly expressed, and apoptosis occurs when GSDME is expressed at low levels in the presence of chemotherapy drugs 44. In addition, other scholars have shown that GSDME can be cleaved by granase B (GZMB) to induce pyroptosis 43. Yersinia YopJ protein can cause the cleavage of GSDMD through caspase-8, thus inducing pyroptosis 45,46-48. Additional studies have shown that spase-6 activates the NLRP3 inflammasome by enhancing the interaction between serine/tryptophan protein kinase 3 and Z-DNA binding protein 1, which in turn activates caspase-1-mediated pyroptosis 44. In addition to GSDMD and GSDME, GSDMA/B/C also plays an important role in membrane perforation and pyroptosis 19, 49, 50. It has been found that granase A in cytotoxic lymphocytes can cleave GSDMB and cause pyroptosis. The GSDMB-NT lacks a specific connection area and cannot be cleaved by caspase-1/4/5/11 but can be recognized and cleaved by caspase-3/6/7 51. Therefore, the role of GSDMB in pyroptosis is still controversial. GSDMA-NT, GSDMD-NT and GSDME-NT showed similar pore-forming activities 19. However, the exact mechanism has not yet been reported. GSDMC has been shown to play a role in pyroptosis. GSDMC was first shown to be highly expressed in metastatic melanoma and is also known as melano-derived leucine zipp-containing extranuclear factor (MLZE) 50. GSDMC can be cleaved by caspase-8. The presence of programmed death ligand 1 (PD-L1), macrophage-derived TNF-α, antibiotics, or chemotherapy can induce pyroptosis by the caspase-8/GSDMC pathway, and TNF-α can induce pyroptosis through GSDMC in MDA-MB-231 breast cancer cells 48.

## Pyroptosis and tumors

### Pyroptosis observed in various tumors

In HCC, researchers have shown that NLRP3 and ASC expression was significantly downregulated, which was negatively correlated with the pathological grade and clinical stage of HCC 52. The expression of caspase-1 was significantly decreased in HCC tissues, and caspase-1, IL-1β and IL-18 in HCC tissues were lower than those in paracancerous tissues. Euxanthone inhibits the development of HCC by inducing pyroptosis 53, 54. In HCC tissues, the expression of DFNA5/GSDME was lower than that in normal tissues, the expression of DFNA5/GSDME was upregulated, and cell proliferation was inhibited 55. Pancreatic ductal adenocarcinoma (PDAC) is a highly malignant tumor, and the therapeutic effect is still not ideal. The expression of STE20-like kinase 1 (MST1) in PDAC is decreased. Restoring MST1 expression can lead to increased PDAC cell death, and caspase-1-mediated pyroptosis can inhibit proliferation, invasion and metastasis through ROS induction 56. In breast cancer, Wu et al. showed that the expression levels of caspase-1, IL-1 and GSDMD were negatively correlated with tumor grade, size, stage, and risk of death 57. Docosahexaenoic acid (DHA) inhibits breast cancer, and when it is added to the breast cancer cell line MDA-MB-231, caspase-1 and GSDMD activities are enhanced, and pyroptosis occurs, which manifests as increased IL-1β secretion and membrane perforation 58. In the development of esophageal cancer, alcohol consumption has been shown to inhibit caspase-1 and promote IL-18 and IL-1β and has been associated with pyroptosis 59. Alcohol consumption exacerbates the course of esophagitis through pyroptosis. Gastroesophageal reflux disease (GERD) increases the risk of Barret's esophagus and esophageal cancer due to long-term exposure of the esophageal epithelium, to damage to the esophageal mucosa and chronic inflammation stimulated by alcohol consumption 60, 61. In addition, LPS can also play a role in the occurrence of esophageal cancer by inducing pyroptosis via the noncanonial pathway 62. In gastric cancer, studies have shown that a reduction in GSDME expression promotes the progression of gastric cancer 17. During chemotherapy, GSDME can be activated by caspase-3 to induce pyroptosis in gastric cancer cells 45, 63. In addition, pyroptosis plays a role in the occurrence of tumors and the inhibitory effect of drugs on tumors in many other cancers, such as lung cancer 64, glioma 16, 35, and ovarian cancer 65.

### Pyroptosis and tumor immunity interact

Pyroptosis occurs in normal cells, which may change the microenvironment. The inflammatory microenvironment can help accelerate the immune escape of tumors and have a protumor effect 66. Long-term tissue or cell exposure to an inflammatory environment can increase the risk of cancer. We have found that cancer cells can adapt to the immune microenvironment, undergo immune escape from immune attack, and adjust the progression of the primary tumor and metastasis. Pyroptosis provides a chronic inflammatory environment for tumorigenesis to promote tumors through inflammasomes, supporting the tumor microenvironment and the production of inflammatory cytokines 67. Inflammasomes regulate the tumor immune microenvironment and cell death, and the intestinal microbiota plays an important role in tumorigenesis and metastasis 68. Inflammasomes can be clinically used as prognostic markers for cancer patients 33, 68. In addition, some treatments can stimulate the immune system and induce pyroptosis in tumor cells 69. The role of pyroptosis in tumorigenesis and metastasis is related to many factors, including the activation of proto-oncogenes, the inactivation of tumor suppressor genes, changes in the immune microenvironment, oxidative stress and chronic inflammation. Activation of the pyroptosis pathway results in the release of inflammatory mediators such as IL-1 and IL-18 into the microenvironment, which can promote cancer transformation in tissue. Researchers showed that mice lacking active inflammasomes were more likely to successfully develop colitis-associated colon cancer than wild-type mice 70. These results suggest that pyroptosis may play different roles in promoting and inhibiting the growth of different tumor cells. The specific mechanism of pyroptosis and its relationship with tumors still need further study. The occurrence and development of tumors cannot be separated from the immune escape of tumor cells, and the reactivation and maintenance of the immune response to tumors can play a role in the control and elimination of tumors. Cancer cells have immunogenicity, and targeting the immunogenicity of cancer cells is a common tumor immunotherapy 71, 72. Because inflammation, especially persistent chronic inflammation, plays an important role in the development, progression, angiogenesis and metastasis of cancer 73, many tumors are secondary to chronic inflammation, which is mediated by M1 macrophages, natural killer cells, and CD8+ T cells 74. Tumor cells can also recruit specific subsets of immune cells to participate in tumor suppression, including myeloid suppressor cells (MDSCs), M2 macrophages, and regulatory T cells 75, 76. However, in tumors expressing GSDME, pyroptosis produces DAMPs that can recruit immune cells to the tumor microenvironment, and GSDME expression greatly increases the number of tumor-infiltrating lymphocytes (TILs) and macrophage phagocytosis 77. In the absence of GSDME, caspase-3 activation can lead to apoptosis, which is characterized by cell contraction and plasma membrane blistering. Apoptotic cells are cleared by neighboring phagocytes before they lose integrity and are necrotic cells with low immunogenicity. The activation of caspase-3 when GSDME is highly expressed can induce pyroptosis. Cell swelling and rapid rupture of the plasma membrane indirectly increase the recruitment of immune cells and their role in tumor suppression by promoting an inflammatory and potentially immunogenic tumor environment. Due to pyroptosis, tumor immunity is promoted and tumor growth is inhibited. Although GSDME is expressed in only a small number of tumor cells, a small number of tumor cells undergoing pyroptosis are sufficient to modulate the tumor immune microenvironment and activate a powerful T-cell-mediated antitumor immune response 78. In immunodeficient mice, tumor suppression by GSDME disappeared due to a lack of NK cells and CD8+ killer T cells, suggesting that this inhibition was dependent on these two immune cell types 43. Therefore, research and development of antitumor drugs can be guided by the idea of tumor immunity and the tumor microenvironment, and the expression of GSDM family proteins can become a potential marker of tumor immunotherapy.

Pyroptosis is a double-edged sword that plays an important role in tumorigenesis and antitumor immunity at all stages of tumor development. Whether it promotes or inhibits tumors depends on the tumor type, host inflammatory state, immunity, and related effector molecules. When tumor cells undergo pyroptosis, the inflammatory factors IL-1β and IL-18 are released, and these inflammatory factors can promote and fight tumors 76. Studies have shown that the expression of IL-1β in tumors is higher than that in normal tissues, and this factor can promote the growth, invasion and metastasis of tumor cells, which is negatively correlated with prognosis 79. The expression of IL-18 in tumors is also increased and negatively correlated with prognosis. High expression of IL-18 also promotes the biological behavior of tumors 80-82. However, IL-18 has dual effects. This factor also regulates the immune response and inhibits tumor progression through the recruitment of NK cells, T cells and monocytes 82, 83. The tumor-promoting and tumor-suppressive effects of chronic inflammation induced by pyroptosis are similar mechanistically, so the timing, level and composition of pyroptosis induction need to be closely controlled.

### Clinical anti-tumor strategy and pyroptosis

At present, the clinical anti-tumor treatment is still mainly surgery, radiotherapy and chemotherapy, as well as the recently emerging immunocheckpoint inhibitors, targeted therapy, etc. Recent studies have also found that some of the anti-tumor treatment methods used in clinical practice also play an anti-tumor role by inducing pyroptosis and changing the tumor microenvironment to promote tumor immunity. The following content mainly systematically combs the interaction between clinical anti-tumor strategies and pyroptosis.

Surgery has always been an important means of solid tumor treatment. At present, it is still the main treatment for solid tumors. Although the effect of surgery on tumor cell pyroptosis is rarely mentioned by scholars, recent studies have found the relationship between surgery and tumor immunity. Immunosuppression after surgery is reported by scholars 84. After tissue damage, DAMP is released, causing cell pyroptosis and inflammatory environment, and then recruiting immunosuppressive cells, such as MDSC, M2 macrophages, etc. Therefore, the impact of surgery on human body may be unfavorable from the perspective of anti-tumor immunity. Eliminating the activation of the pyroptosis pathway caused by surgery can reduce the immunosuppression caused thereby.

A variety of chemotherapeutic drugs have been proved to be able to induce tumor cell pyroptosis, including cisplatin, paclitaxel, 5-FU, lobaplatin, etc. Chemotherapy induced pyroptosis is often caused by activation of GSDME pathway. In the lung cancer cell line A549, researchers showed that both cisplatin and paclitaxel could induce pyroptosis in tumor cells through the caspase-3/GSDME pathway, and cisplatin acts stronger than paclitaxel 85. High levels of GSDME can transform apoptosis into pyroptosis. Some studies have shown that the chemotherapy drug lobaplatin can induce pyroptosis in cervical cancer cells through GSDME 86. This effect is achieved through activation of caspase-3/9 by the ROS/JNK/BAX mitochondrial apoptosis pathway. Similarly, lobaplatin can also induce pyroptosis in colorectal cancer cells through this pathway 87. In gastric cancer, 5-FU induces pyroptosis in gastric cancer cells through GSDME rather than GSDMD 63. In GSDME^+/+^ mice, cisplatin or 5-FU can cause severe intestinal injury and immune cell infiltration, while GSDME^-/-^ mice have fewer signs of injury. Moreover, GSDME knockout can also reduce lung injury in response to cisplatin or bleomycin in mice 88. These effects suggest that GSDME-induced pyroptosis is associated with chemotherapy side effects. These results also suggest that inducing pyroptosis in tumor cells is a possible alternative strategy for tumor therapy.

Many drugs in addition to chemotherapy drugs exert antitumor effects through GSDME-mediated tumor cell pyroptosis 45. However, researchers showed that only approximately 1 in 10 human tumor cells have high levels of GSDME compared with 3 in 5 primary cells. After chemotherapy, tumor cells with high levels of GSDME can undergo pyroptosis, and GSDME-mediated pyroptosis is associated with toxicity and side effects caused by chemotherapy drugs 45. Some studies have shown that GSDME is epigenetically silenced in several cancers, such as gastric cancer, colorectal cancer and breast cancer, and is considered a tumor suppressor gene. This gene may be epigenetically inactivated through methylation, and its promoter is hypermethylated in several cancers, demonstrating that the main form of gene silencing is hypermethylation 89, 90. GSDME methylation is considered a promising biomarker for cancer detection. In addition, endogenous GSDME expression is closely related to the response to chemotherapy or immunotherapy. It has been reported that low GSDME expression can impair antitumor efficacy, while increasing GSDME expression levels can improve the efficacy of antitumor therapy 45, 85, 91.

Many cancer patients receive radiotherapy, which destroys tumor cells through high-energy radiation. Cao, W found that ionizing radiation can trigger tumor immunity by inducing GSDME mediated pyroptosis in tumors 92. It was found that the fragmentation of GSDME occurred in a dose-dependent and event-dependent manner, and all kinds of irradiation could induce cell death. In addition, cytotoxic T cells and cytokine release appear after pyroptosis. Radiation causes the death of immunogenic cells and promotes anti-tumor immunity. Radiotherapy can directly destroy DNA and kill cancer cells. After recognizing DNA fragments through AIM2 receptor, the inflammasome will be activated and induce pyroptosis, and the release of inflammatory mediators will increase tumor infiltrating immune cells, thus making the “cold” tumor “hot” in immunology 93.

During the process of tumor development, immune escape occurs. Restoring the visibility of tumor antigens to the immune system is essential to inhibiting immune escape and increasing tumor immune activation. Immune checkpoint inhibitors promote a targeted immune response to neoplastic antigens. Pyroptosis may play an important role in this treatment. Programmed death-1 (PD-1) and programmed death ligand-1 (PDL-1) are immune checkpoint regulators that are targets of widely used immune checkpoint inhibitors 94. Gradually, researchers found a relationship between pyroptosis and these factors. Currently, the PD-1 and PDL-1 pathways are found to be important in cancer immunosuppression 95. Mien-chie Hung et al first reported that PD-L1 interacts with P-Y705-STAT3 and then induces the nuclear translocation of PD-L1 under hypoxia. The function of nuclear PD-L1 at the transcriptional level contributes to the expression of GSDMC. Then the apoptosis induced by TNFα was transformed into pyroptosis 48. Clinical trials have shown that PD-L1 inhibitors combined with chemotherapy or radiation can kill tumor cells via pyroptosis, and there is improved survival compared to patients treated with PDL-1 inhibitors alone 96. Combination therapy increases the sensitivity of breast cancer cells to PD-1/PDL-1 inhibitors due to inflammation caused by pyroptosis in the tumor immune environment. In the presence of GSDME, caspase-3 activation leads to pyroptosis, and the formation of an inflammatory environment can increase the recruitment of immune cells 43. Trimethylamine N-oxide** (**TMAO) can induce GSDME-mediated pyroptosis in breast cancer cells, and TMAO in combination with PD-1 can increase the antitumor activity of PD-1 alone. Researchers found that TMAO is more abundant in tumors with an activated immune microenvironment and that TMAO can enhance CD8+ T-cell-mediated antitumor immunity by inducing pyroptosis of tumor cells through activation of ER stress kinase PERK 97. If PD-1 or PDL-1 inhibitor therapy is the initial tumor treatment, inflammation caused by pyroptosis further potentiates the effects. These studies provide a theoretical basis for the combination of PD-1/PDL1 inhibitors and other antitumor therapies. The inflammatory state of the tumor microenvironment has an impact on the response to immune checkpoint inhibitor therapy, which changes the tumor microenvironment and the role of lymphocytes in the tumor by triggering pyroptosis and transforming tumors more sensitive immunologically. Thermalization of the immune response is a complex process that directly regulates the innate immune response by releasing DAMPs and inflammatory factors, enhancing the recruitment of adaptive immune cells, and increasing antigen presentation and TLR activation, thereby amplifying the immune response.

Chimeric antigen receptor T (CAR-T) cells have been used to treat hematological malignancies and have achieved good results. However, cytokine release syndrome (CRS) is a serious side effect of this technology. The release of granase B by CAR-T cells may lead to pyroptosis by activating the caspase-3/GSDME pathway 42, and GSDME knockout eliminates CRS. In addition, the number of perforin/granzyme B in CAR-T cells, rather than existing CD8+T cells, will induce GSDME mediated target cell pyroptosis 42. Recent studies have also shown that the synergetic effect of TNF and INF- γ will form a positive feedback loop between inflammatory cell death and cytokine release, and then drive CRS 98. These results indicate the multiple roles of pyroptosis in tumor immunotherapy, which has corresponding clinical significance.

In addition to immune checkpoint inhibitors and adoptive T-cell therapies, immunogenic cell death has received increasing attention in the field of tumor immunity 99. Pyroptotic cells can also exert their immunogenic effects 100, and IL-1β and IL-18 released by pyroptotic cells, as well as various DAMPs, can recruit immune cells such as dendritic cells or macrophages to engulf pyroptotic cells. Mature dendritic cells present their antigens to tumor-specific toxic T cells to kill tumors 101.

Wangmeng and colleagues found that TBD-3C, a membrane targeted photosensitizer with aggregation induced emission (AIE) characteristics, triggered cell charring through photodynamic therapy (PTD) to cause cancer immunotherapy. TBD-3C induced cell pyroptosis can stimulate M1 polarization of macrophages, lead to dendritic cell (DC) maturation, and activate CD8+cytotoxic T lymphocytes (CTL). It can not only inhibit the growth of primary pancreatic cancer, but also attack distant tumors 102. Nanodrug is a new tumor treatment method combining traditional drugs and nanotechnology. It is a carrier of controlled release chemotherapy drugs, which can directly deliver chemotherapy drugs to targeted cancer cells, reduce their accumulation in normal cells and tissues, and reduce the side effects of chemotherapy. Nanodrugs can play an anti-tumor role by inducing cell pyroptosis 103, 104. Researchers have shown that treating A549 cells with zinc oxide nanoparticles (Zn-ONPs) induced IL-1β release and caspase-1 activation and increased LDH release, indicating pyroptosis in A549 cells 105. LipoDDP is a tumor targeting nano liposome carrying cisplatin. DAC is a DNA methyltransferase (DNMT) inhibitor, which can inhibit the methylation of GSDME in tumor cells. LipoDDP combined with DAC can utilize liposomes and activate caspase-3 mediated cell death, and can induce immune response, thereby inhibiting the proliferation and metastasis of tumor cells 106. The combination of photodynamic therapy and nanomedicine as biomimetic nanoparticles can induce both cell pyroptosis and systemic anti-tumor immunity 104, 107, 108.

Small molecule targeted drugs are one of the rapidly developing methods to treat cancer in recent years. Some targeted drugs have been found to induce tumor cell pyroptosis. Val-boroPro induces pyroptosis in primary acute myeloid leukemia (AML) cells by activating the inflammasome sensor protein CARD8, which in turn activates procaspase-1 109. A study in melanoma confirmed that the combination of BRAFi and MEKi could have an antitumor effect through GSDME induced pyroptosis 77, DDP combined with BI2536 (PLK1 kinase inhibitor) can cause esophageal cancer cell pyroptosis^110^. Among the discovered phenomenon of tumor cell pyroptosis induced by targeted drugs, caspase3/GSDME pathway is the most common pathway of targeted drug induced pyroptosis.

The anti-tumor drugs and therapies mentioned above, including new treatment methods such as nano drugs and photodynamic therapy, can induce pyroptosis to play an anti-tumor effect. Other natural compounds and some canonicalal drugs can also promote pyroptosis of tumor cells. Dobrin et al. stimulated triple-negative breast cancer cells with ivermectin, and the pannexin-1 pathway was activated, inducing P2X4/P2X7 receptor overexpression, ATP release, and ultimately pyroptosis 111. Many other drugs, such as metformin, anthocyanin, and DHA, can induce GSDMD mediated pyroptosis in various cancers 58, 112, 113. Pyroptosis widely occur within the tumor cells, a variety of antitumor method, including surgery, radiotherapy, chemotherapy and immune checkpoint inhibitor gradually developed in recent years, small molecular targeted drugs, photodynamic therapy and drugs, as well as some natural compounds, such as traditional Chinese medicine preparation can induce tumor cells pyroptosis and perform antitumor activities.

## Conclusion

The research on pyroptosis has made great progress in the past few years. In this review, we focus on the molecular mechanism of pyroptosis. Pyroptosis is a new type of programmed cell death that is accompanied by inflammation and has been shown to be associated with many diseases, including tumors and atherosclerosis. The relationship between pyroptosis and tumors, pyroptosis and immunity, and its clinical application has become a hot topic. It is of great significance to reveal the role of pyroptosis in many diseases, especially tumors. In particular, in the field of the relationship between pyroptosis and tumors, especially tumor immunity, there are still many questions to be answered about the specific mechanis. Pyroptosis plays different roles in different types of tumors and plays both protumoral and antitumoral roles. Multiple mechanisms have been known to regulate pyroptosis. Various stimuli, to the formation of inflammatory complexes, and to pyroptosis signal transduction, have extremely complex regulatory mechanisms. These regulatory mechanisms not only affect the occurrence of pyroptosis, but also affect the immune response in the extracellular environment. Further study of the relationship between tumors and pyroptosis would be helpful in developing new antitumor therapies, as well as prompt new ideas for the fight against the difficult process of cancers and has great clinical prospects.

## Figures and Tables

**Figure 1:**
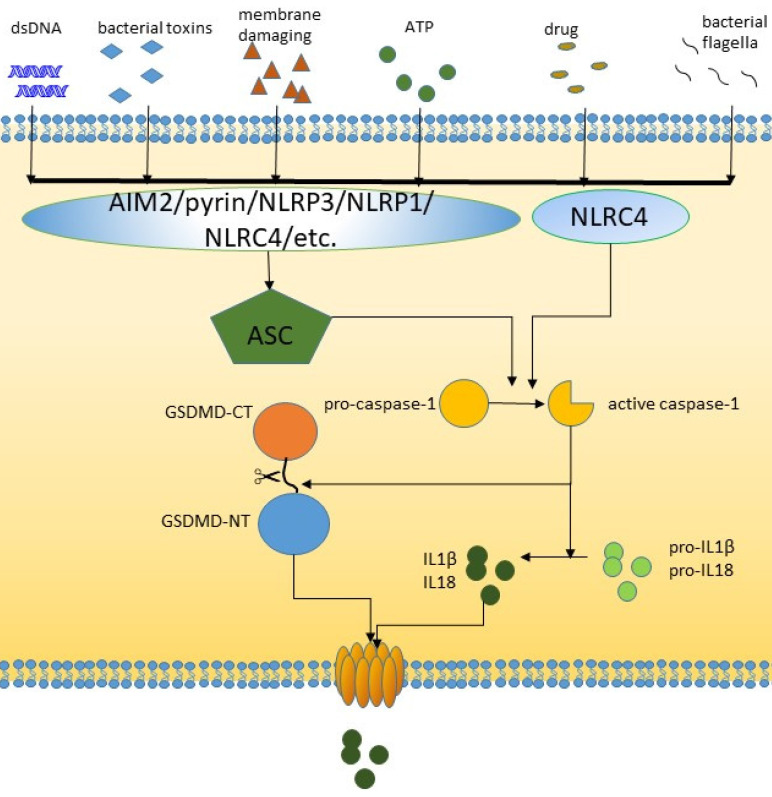
The canonical pathway of pyroptosis.

**Figure 2:**
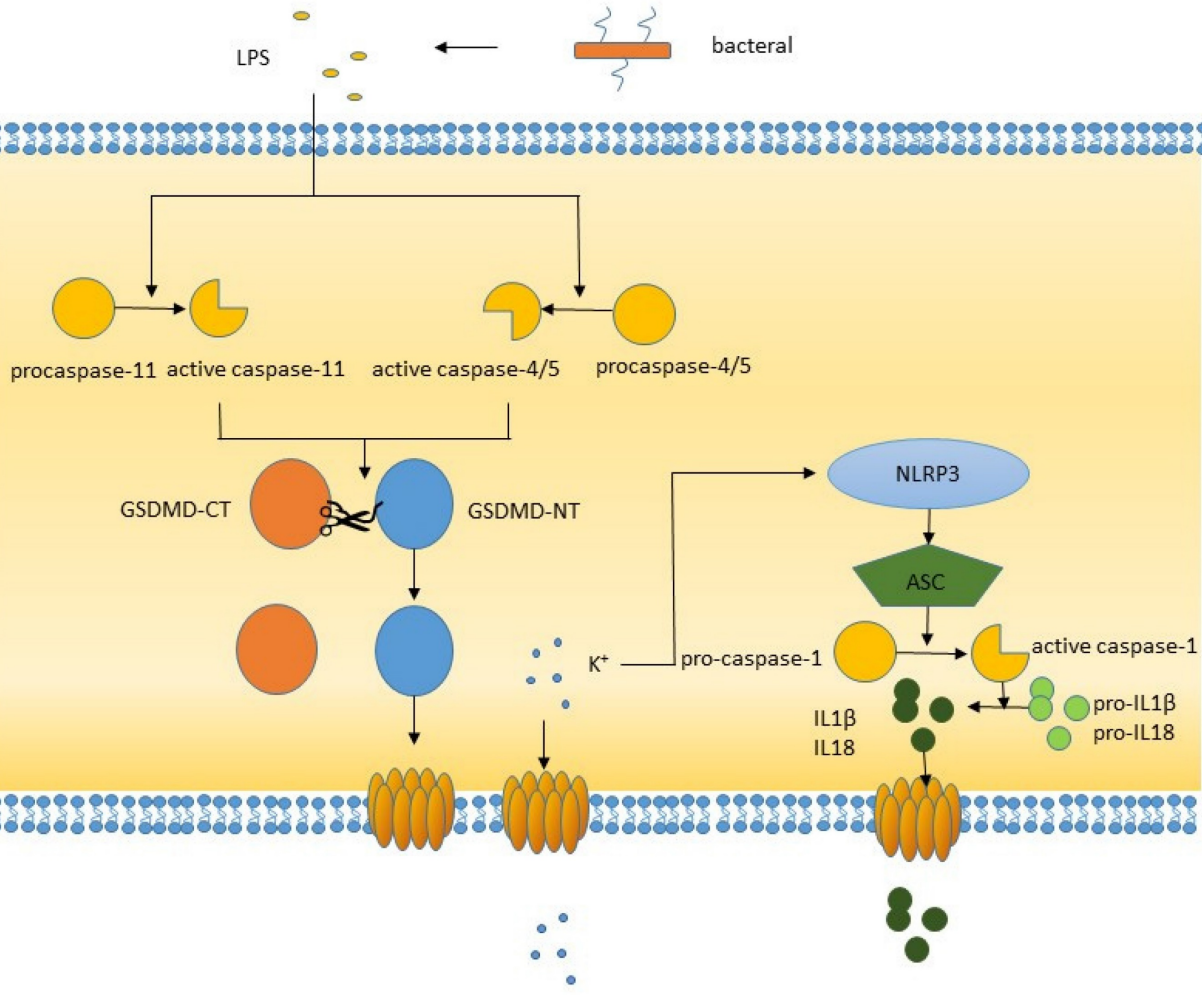
The non-canonical pathway of pyroptosis.

**Figure 3:**
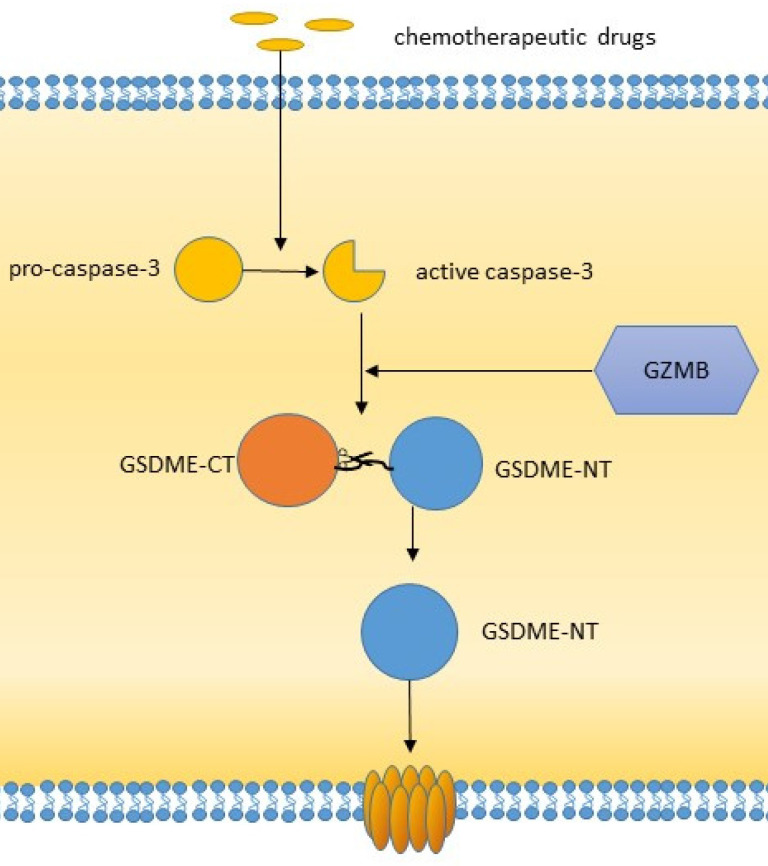
The caspase3/GSDME pathway of pyroptosis.

**Figure 4:**
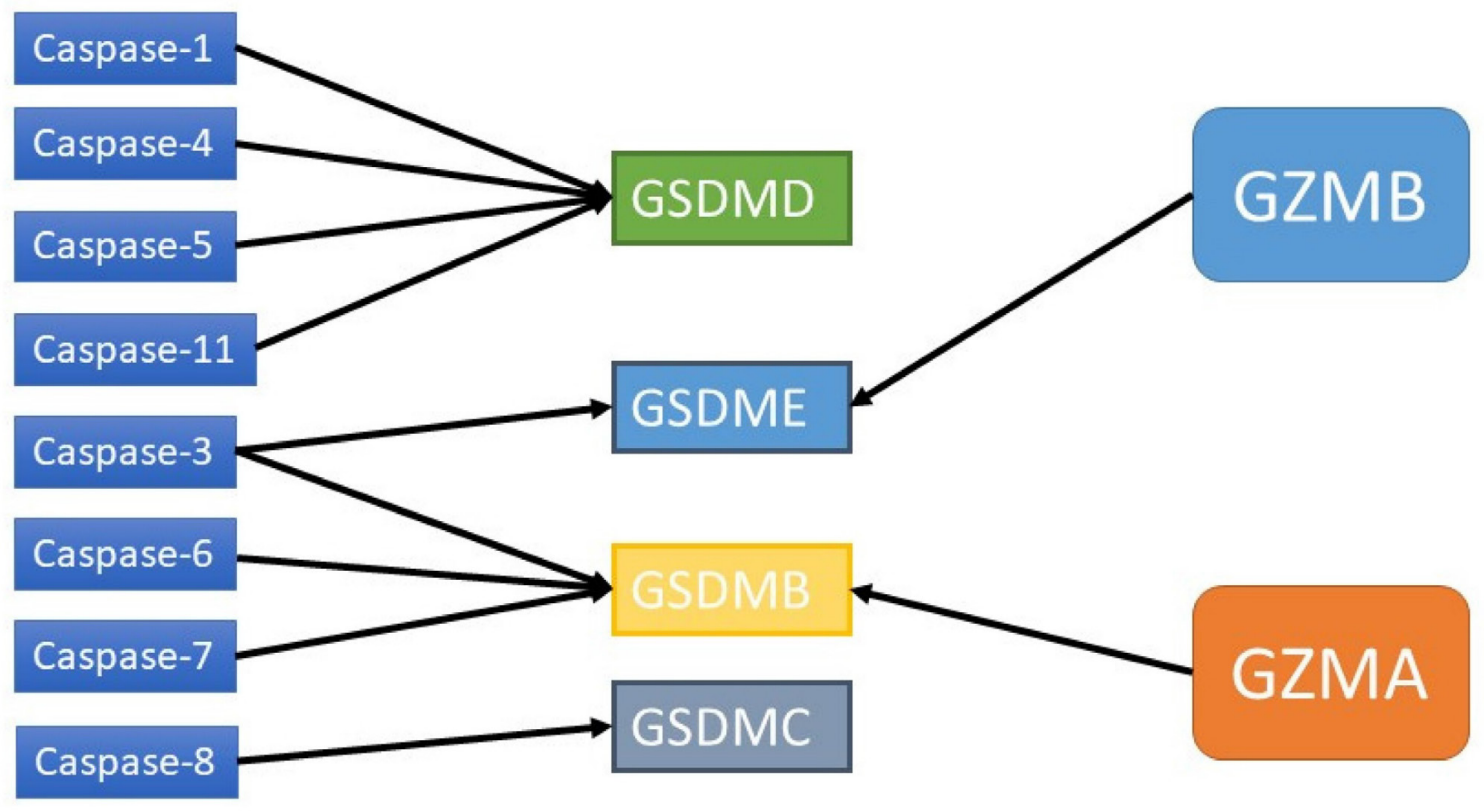
Caspases and GZMs that activate GSDMs.

**Table 1:** Pyroptosis and mechanism in different cancer cells

Cancer type	The role of pyroptosis	Ref
HCC	Down regulation of NLRP3 and ASC in tumor tissue	52
HCC	Down regulation of caspase-1 in tumor tissue	53,54
HCC	Down regulation of GSDME in tumor tissue	55
PDAC	Down regulation of MST1 which contributes to inhibition of pyroptosis	56
Breast cancer	The expression of caspase-1,GSDMD is negatively correlated with tumor malignancy and risk of death	57
Breast cancer	DHA inhibits cancer via caspase-1/GSDMD pathway	58
Breast cancer	TMAO combined with PD-1 inhibitor increase antitumor function by inducing GSDME mediated pathway	97
Esophageal cancer	LPS induced noncanonical pathway of pyroptosis	62
Gastric cancer	Reduction of GSDME promotes cancer progression	17
Gastric cancer	Chemotherapy induce pyroptosis via caspase3/GSMDE pathway	45,63
Lung cancer	Cisplatin and paclitaxel induce pyroptosis via caspase3/GSDMA pathway	85
Cervical cancer	Lobaplatin induces pyroptosis via caspase3/GSDME pathway	86
